# Modeling Impact of Word of Mouth and E-Government on Online Social Presence during COVID-19 Outbreak: A Multi-Mediation Approach

**DOI:** 10.3390/ijerph17082954

**Published:** 2020-04-24

**Authors:** Ammar Yasir, Xiaojian Hu, Munir Ahmad, Abdul Rauf, Jingwen Shi, Saba Ali Nasir

**Affiliations:** 1 School of Management, Hefei University of Technology, Hefei 230009, China; 2016010121@mail.hfut.edu.cn or; 2 School of Economics, Zhejiang University, Hangzhou 310058, China; munirahmad@zju.edu.cn; 3 School of Management Science and Engineering, Nanjing University of Information Science and Technology (NUIST), No. 219 Ningliu Road, Nanjing 211189, China; abdulrauf@seu.edu.cn; 4 School of Management, Anhui University of Science and Technology, Huainan 2320012, China; shijingwen20@gmail.com; 5 School of Business, Anhui University, Hefei 230009, China; sabaalinasir7@gmail.com

**Keywords:** 2019-nCoV-WOM, epidemic outbreak, quarantine, social presence theory, role of E-government, epidemic protection

## Abstract

Although social presence plays an essential role under general conditions, its role becomes significant for societal protection during the quarantine period in epidemic outbreak. In this study, we attempted to identify the role of E-government and COVID-19 word of mouth in terms of their direct impact on online social presence during the outbreak as well as their impacts mediated by epidemic protection and attitudes toward epidemic outbreaks. For this purpose, a unique multi-mediation model is proposed to provide a new direction for research in the field of epidemic outbreaks and their control. Through random sampling, an online survey was conducted and data from 683participants were analyzed. Partial least squares structural equation modeling was used to test the relationships between the variables of interest. The study results revealed that the roles of E-government and COVID-19 word of mouth are positively related to online social presence during the outbreak. Epidemic protection and attitude toward epidemic outbreak were found to positively moderate the impact of the role of E-government and COVID-19 word of mouth on online social presence during the outbreak. The key findings of this study have both practical and academic implications.

## 1. Introduction

The flow of information in any matter is important and can considerably impact the situation during an epidemic outbreak. The role of e-government is also essential in any situation related to health protection, especially during an outbreak period.

**Figure 1:**
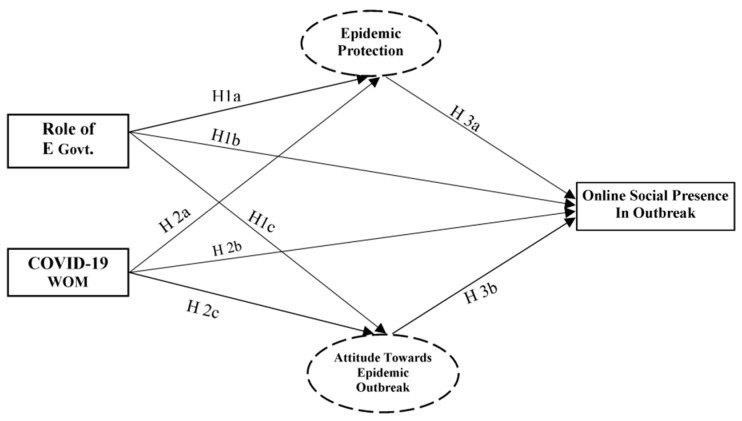
Conceptual model.

E-government (E-Govt) is defined as “the use of information technologies that have the ability to transform relations with citizens, businesses, and other arms of government” [1]. Keeping people calm and focused is necessary during an epidemic and its quarantine period. If people are quarantined, the roles of E-Govt and word of mouth (WOM), especially message sharing through social media, increase in importance. Now, with the current COVID-19 outbreak, people are under quarantine until further notice, with many countries experiencing epidemic outbreaks. People have more time to use social media, which could be a source of rumors, anxiety, and, most important, knowledge for health protection. This phenomenon encouraged us to formulate a theoretical model based on social presence theory and the role of social media to help examine safety and participation in quarantine. The conceptual model is presented in Figure 1.

Word of mouth can be defined as “the intention to share a certain story using one’s own social media account” [2]. Many researchers proved that communication has more of an impact through social media with regards to different health issues, and message sharing was found to be the best predictor of emotive and passionate response [3]. Online health information is regularly updated [4]. Nowadays, universal and worldwide flow cannot be ignored [5] and search engines are the most effective method to find information [6]. The Internet is also a useful source for people in the medical field for discussions and interactions about medical issues [7]. A nine-year big data study showed that the use of computers projected emotive improvement [8]. From the health protection point of view, many factors were found to affect the dispersal of cancer information, including anxiety, courage, anticipation, and sharing of experience [9].

A study proved that the gestation time of infection (corona virus) was assessed at about 2–14 days [7]. So, a research question was raised about protection during an epidemic outbreak. Social media campaigns with complete research frameworks with theoretical and statistical support for these time periods are essentially to protect society during emergencies. To address this need, our research model includes the factors that trigger safety campaigns and the main effects (direct) of direct relations and the association of mediators on online social presence.

The literature shows that for different disease management programmers, among health experts, the most important aspects are self-value, the individual’s reputation [10], and sharing their familiarity and information. However, this issue is seldom observed within a framework of online health communities [10] because, with the spread of disease contacts, heterogeneity is critical [11]. Public health moved to a wider political regime from health-dedicated management in 2013 in England as public health functions were relocated to local management from the National Health Service (NHS) [12]. Acceptance of a meningococcal vaccine was unidentified by inspiration of schools [13].

By keeping an individual’s record, doctors can assess body mass index (BMI) and threats to health more accurately and precisely. Sharing opinions and views on actual proceedings is easy with the help of the Internet [14]. Paying attention to the sharing of data determines self-assurance [15].

In the case of failure, specifically in Nepal [16], a lack of education was observed in practice in community-based health insurance. The authors found that people mostly hold back from taking preventive and measure son time on reporting as few first reported cases in any disease outbreak. When they can see more danger, they might become more careful.

In rural areas, maternal and child health (MCH) systems are progressing because the government has formed different partnerships and associations at different levels [17].In countries other than China, secrecy and trust were found to be hindrances to the use of social media [18]. Some countries do not allow people to post health information on social media without authentic reports [19]. Online communal support is also affected by online communication of health professionals with patients [20] and the use of online technology for societal help [15]. We cannot ignore that fear, gossip, and rumors are spread online by some medical experts [21]. The above literature shows the role of social media in spreading information, although a small number of rumors may also be spread through society.

Loyalty and devotion to one’s country is emotive behavior, especially in republic countries [22]. The Chinese nation is one of most patriotic nations in the world. Our study area, Wuhan, is considered the Chicago of China due to its heavy road traffic, and the population of Wuhan is more than 11 million [23]. Anhui province is next to Wuhan, containing the second city reporting patients with COVID-19, after Wuhan. Computers do not have a direct impact on physical health activities but play an intellectual role in online social awareness. The reliability of messages and information along with the trustworthiness of websites are important with regards to health [24]. However, users’ views about their health conditions were the same after using an app as before using the app [25]. Compared to men, women post more statements on social media about health problems online and argue more with physicians’ online statements [26]. This shows that women are more emotional about health conditions, especially during an epidemic outbreak.

Evidence was provided that rumors are spreading during the COVID-19 outbreak in other countries, but in China, people were likely to improve their emotions in the fight against COVID-19 and stay strong during the outbreak period by sharing positive posts on social media. As of the date of writing, 9 March 2020, we have been in quarantine since20 January2020, having direct experience with both normal life and quarantine.

On social media, the importance of being online, especially during an epidemic outbreak, and the role of E-Govt in the quarantine period shown in previous research, urged us to explore this phenomenon on a broader basis. Positive awareness through the effects of COVID-19 word of mouth (2019-nCoV-WOM) and E-Govt in the epidemic period has not been sufficiently explored. Regardless of the important roles of 2019-nCoV-WOM and E-Govt in the epidemic outbreak, quantity of research is lacking. Although online social presence is important at this time, scholars have not explored it sufficiently. From the literature, questions were raised whether online social presence increased the ability to obtain information about the safety measures and, with 24 h of free time to use social media, if people might be more willing to spread safety information. On this basis, we tried to explore the direct effect of 2019-nCoV-WOM and E-Govt on online social presence, and tried to fill this gap using a cohesive methodology to identify the mediating effect of attitude toward epidemic outbreak and epidemic protection on online social presence. We used five constructs (2019-nCoV-WOM, role of E-Govt, attitude toward epidemic outbreak, epidemic protection, and online social presence in the outbreak) with a conceptual multi-mediation model. We explored a distinctive approach to answer two questions: Is there any mediating effect of attitude toward epidemic outbreak and epidemic protection on online social presence? What is the best possible combination for the government to increase people’s willingness to participate in quarantine with a psychological perspective?

In the next section, we discuss our research model. Based on the analysis, we discuss our study results in Section 4. Finally, consequences and practical implication of our research are given and future research is suggested in Section 7.

To fill these research gaps, we provide a new direction for research about epidemic outbreaks by discussing the role of E-Govt and the effect of 2019-nCoV-WOMin relation to the use of social media and their mediating effect on long-term outbreaks. Our research questions (RQs) were:

RQ1: What is the association between social media, epidemic protection messages, and online social presence?

RQ2: Is there any mediating association between 2019-nCoV-WOM and the role of E-Govt in online social presence?

## 2. Literature Review and Hypothesis Development

### 2.1. Role of E-Govt 

People have perceptions about their government playing a role during an epidemic condition. Perceptions are heightened during an outbreak period, as people are concerned about their protection, and their attitude toward an epidemic outbreak depends on this protection, to some extent. During a quarantine period, as people are isolated, the perceived role of E-Govt may be increased and people might be motivated to play a role in epidemic protection after watching the involvement of E-Govt. In our questionnaire, we asked about trust in E-Govt, which might provide motivation to play an important role in epidemic protection.

The literature shows that the role of government, especially governmental strategies and alertness messages, in any outbreak is progressively enhanced [27]. Communication and interaction with the government has increased in last few years, as the government is interested in engaging its people through social media [28]. The abovementioned literature justifies that the government understands people’s perceptions about the role of E-Govt.

People involved in policy-making are also inspired by social media [29]. Factors involved in sharing information are not described in this literature [30]. Attitudes and awareness is connected to the supposed efficiency of policy-making [31]. Improvement and development in Chinese e-waste has been seen in the last six years [32].This might enhance the people perceptions about E-Govt playing a role in an epidemic outbreak. In Canada, social media was used as tool for facilitation of consumers by government officials [33]. In Latin America, research on E-Govt is increasing and conflicting with what is occurring in the world [34]. 

One reason we involved E-Govt and online social presence in our research model is that a noteworthy difference was identified the in analysis of awareness about the use of mobile phones to seek health information during critical times [35]. Awareness promotion plays an important role in burn cases [36]. Universal and global research is needed in community health for the use of social media in E-Govt [37].

Our study variables included capability of isolation, quarantine compartment, and interaction methods, which are responsible for occupancy rate isolation [38]. To stop the transfer of infectious disease, isolating infected people from healthy ones is vital [39]. The biggest outbreak of coronavirus, the Middle East respiratory syndrome (MERS), occurred in South Korea in 2015. The main spread of this virus occurred in South Korea from and out of hospitals [40]. As an independent variable, we added role of E-government in our study, because studies were lacking on the emotional influence of MERS outbreaks [41].

Research showed that administration, media, and celebrities play important roles in health promotion on Twitter [42] because people are more attracted to celebrities. Recommendations that are logically expressed have a strong impact on public [43]. Many governments are improving their use of social media in health departments and are trying to provide digital services to their people guidance; especially during outbreaks. To increase the theoretical literature on E-Govt in the health sector, we aimed to fill this gap using a systematic review method to analytically evaluate, recognize, and create research evidence for the use of 2019-nCoV-WOMin connection with the role of E-Govt in protection from COVID-19. We also tried to critically evaluate the role of social media and the willingness to undergo a long quarantine period for protection from this epidemic. Here, per the literature, we take this variable of people’s perception (perceived role) of the role of the Chinese government, which was important because the government provided a plan to withstand the outbreak period. Notably, this is the first critical review of the use of 2019-nCoV-WOM and the role of E-Govt in the COVID-19 epidemic (as independent variables).

Given the above literature, we expected that E-Govt plays a considerable role in the perception of epidemic protection and online social presence and in changing public attitudes toward an epidemic outbreak. Therefore, we hypothesized the following:

**Hypothesis 1a (H1a):**  *Role of E-government considerably affects epidemic protection.*

**Hypothesis 1b (H1b):**  *Role of E-government considerably affects online social presence during an outbreak.*

**Hypothesis 1c (H1c):**  *Role of E-government considerably affects attitude toward an epidemic outbreak.*

### 2.2. COVID-19-Word of Mouth

WOM provides new directions to people’s thoughts and views about any condition, and especially during an epidemic outbreak. Nowadays, the top trend is COVID-19 epidemic conditions discussed in news and online social websites.

Many studies in the last 10 years proved that there has been a large increase in the number of people willing to find health information on the Internet. An increase in the number of social profiles providing health information was also observed [44]. Electroencephalography inter-subject correlation (EEG-ISC) was improved by an increase in resilient health communication [45]. The people of Wuhan felt great confidence in sharing their views during data collection during the COVID-19 epidemic outbreak quarantine.

Health promotions broadcast by the media are used to promote awareness. For young Italian people, the messages were mostly about health and the environment [46]. This is significant because health experts have been using it as a motivational tool during the treatment of patients [47] and because histories of patients with similar symptoms are important for predictions [48]. This may be helpful for the emotional treatment of patients in the future. Conversely, the most important aspect of health promotions is increasing people’s patience and keeping them calm during the quarantine period to avoid anxiety. The Chinese government improved public endurance by their emotional awareness through message sharing, which encouraged people to share their views [26]. It is a common for people to first think that social media information is only rumors during an outbreak; as such, people were at risk of being ignorant of health information shared by the government or by individuals.

To provide better and urgent cure, people should know the signs and symptoms of corona viruses. Many symptoms of the novel COVID-19-infected pneumonia (NCIP) have been described by researchers; they include increased body temperature, dry cough, and body pain [49]. The Chinese government promoted awareness among people through social media, so that individuals showing signs of COVID-19would immediately understand and contact medical experts for treatment and minimize further spread of the virus. Only online 2019-nCoV-WOM was used for the quarantine period in China, especially in Wuhan and Anhui; some other countries soon after implemented quarantine measures. This means the Chinese government played a positive role in the safety of people. 

The above-mentioned research demonstrated the importance of online 2019-nCoV-WOM. So, we tried to explore the positive effect of 2019-nCoV-WOM on online social presence in our research using exclusive mediating variables. We expected that 2019-nCoV-WOM not only significantly affects perceptions of epidemic protection in terms of individuals’ online social presence, but also influences changes in attitude toward the epidemic outbreak. So, we hypothesized the following:

**Hypothesis 2a (H2a)**  *COVID-19 word of mouth considerably affects epidemic protection.*

**Hypothesis 2b (H2b):**  *COVID-19 word of mouth considerably affects online social presence.*

**Hypothesis 2c (H2c):**  *COVID-19 word of mouth considerably affects attitude toward the epidemic outbreak.*

### 2.3. Epidemic Protection from COVID-19and Its Mediating Role

Everyone is concerned with their protection in any condition, but particularly during an epidemic outbreak. The protection factor might affect social presence, but as people are isolated during quarantine, this factor changes the effect on online social presence in other ways. 

Individuals’ attitudes toward fitness is boosted by viewing health information posted on Facebook in video form [3]. People’s responses to epidemic protection increase especially during quarantine because they are at home and have more free time. Individuals with prolonged health problems acknowledged caretakers’ guidance more than other adults in the U.S., but they did not obtain physical exercise guidance for good health [50]. The COVID-19 epidemic protection urged people to increase their online social presence to enhance public emotions for epidemic protection.

The empirical studies mentioned above encouraged us to relate epidemic protection as a dependent variable due to obtaining specific information to protect online users during the quarantine period. Here, we took this variable as people’s perception to ensure their safety. China is more conscious about health due to the one-child policy, free Internet to obtain information, and the perception of being safe through obtaining safety measures. Therefore, we hypothesized the following:

**Hypothesis 3a (H3a):**  *Epidemic protection considerably affects online social presence.*

The literature discussed above indicated that assuming the role of E-Govt in online social presence of the public is mediated by epidemic protection and 2019-nCoV-WOM affects online social presence. However, we suspected that 2019-nCoV-WOM is boosted by the psychological perception of epidemic protection. So, we hypothesized the following:

**Hypothesis 3c (H3c):**  *Epidemic protection mediates the association between COVID-19 word of mouth and online social presence.*

**Hypothesis 4b (H4b):**  *Epidemic protection mediates the association between role of E-government and online social presence.*

### 2.4. Attitudes toward Epidemic Outbreak and Its Mediating Role

**Figure 2:**
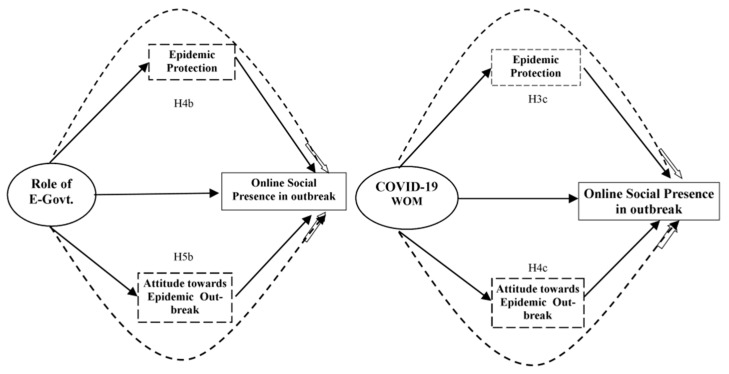
Multi-mediation model.

A study showed that people’s attitudes toward an epidemic outbreak are more influenced by information on social media compared with physical discussion because source reliability has no impact on the health information provided online [51].People obtain information about the outbreak through social media and mobile health apps [26].However, a risk of client secrecy exists because online services are substantially affected by the happiness of clients [52].We took attitudes toward an epidemic outbreak as a mediating effect, meaning that attitudes toward an epidemic outbreak will promote or mediate the relationship of two independent variables and social online presence, as shown in Figure 2.

Research showed that the availability of a vaccine for an epidemic affects people’s attitudes toward the outbreak; for publicizing a vaccine, online sources are important. During epidemics, curiosity about a vaccine is more influenced by publicity, not by the epidemic conditions. However, vaccine uptake is also influenced by the epidemic condition when epidemic increases [53]. Recommendations by doctors, friends, and relatives stimulate people [54] as they discuss their responses to epidemics.

People who are isolated during an epidemic must recognize the importance of protection [55]. Research in Toronto, Canada, proved the willingness of people to participate in studies and the stress experienced by medical experts due to wearing of caring apparatus and being infected by disease in Canada. As these medical experts were involved in attempting to cure a disease about which they had little knowledge, they were worried that they would be next targets when they saw their coworkers becoming sick and dying due to the epidemic [56].

Lack of trust in government was observed in the public in Korea when attempting to control the MERS epidemic [57]. However, our focus was on the effect of 2019-nCoV-WOM and the role of E-Govt to protect the people during long-term outbreak, and to examine the response of people during the quarantine period. We supposed that public attitude toward epidemics affects their online social presence. In China, people are willing to share news and have positive attitudes when sharing the good news about protection. So, we hypothesized the following:

**Hypothesis 3b (H3b):**  *Attitude toward epidemic outbreak considerably affects online social presence.*

The attitude of the public toward a situation impacts their online social presence and affects the role of E-Govt and 2019-nCoV-WOM on online social presence. This means that if the attitude of the public is positive, it mediates role of E-Govt and online social presence. Hence, we hypothesized that:

**Hypothesis 4c (H4c):**  *Attitude toward epidemic outbreak mediates the association between COVID-19 word of mouth and online social presence.*

**Hypothesis 5b (H5b):**  *Attitude toward epidemic outbreak mediates the association between the role of E-government and online social presence.*

### 2.5. Online Social Presence in Epidemic Outbreak

According to social presence theory [58], social presence is about intimacy, feeling of closeness, familiarity, immediacy, and urgency to exchange information and motives in society. Online social presence is important during epidemic outbreak quarantine periods. It not only plays an important role in the coordination of society but also in the creation of motives.

Online social presence is predicted by online streaming, mediating communal television pleasure [26].

Among the magnitudes of social presence (telepresence and social presence), online social presence indirectly plays a role in the mediating direction. Findings encouraged innovative marketing policy through which participation can be optimistic by refining presence fundamentals [59]. People are more attracted toward celebrities, which strengthens online social presence [60]. So, if celebrities are active during outbreak periods, they can motivate people to increase their online social presence.

In addition, human sympathy is apparent on online sites and especially social sites, representing their online social presence and recognizing their interactions and feelings [58,61].

The capability of a platform to deliver personal indications and increase online social presence willingness [59] is interceded by useful commitments [62]. Every website provides specific confidentiality, which is expressively affected by online social presence [63]. Private platforms are facilitated by online social presence [64], so we predicted that they also affect the online response of people in quarantine during epidemics.

Sociability, pleasure, and belief are emotive reactions that reconcile social presence [59]. These three responses were felt across the Chinese nation during the COVID-19 outbreak. People believe that we will overcome this deadly virus and people were seen to be more emotional and social during this pandemic. Online social presence varies from their level of appointment, which reveals intellectual burden [65]. Contribution by people is inspired by their social value [66]. Addiction to social networking sites (SNS) also improves online social presence and increases people’s pleasure when interacting socially, also improving social communication and gratification [26]. Online social presence influences online engagement [67].

## 3. Materials and Methods

### 3.1. Study Area

We included two areas in our study: Wuhan in Hubei (all cities from this province) and Anhui province. Participants of our study were mostly from Wuhan. Anhui (Hefei province) is the nearest city to Wuhan, so we also considered Anhui in our study. We were all in quarantine (except one doctor as a writer for medical terminology association but she is not living in China), directly experiencing the feelings of this situation.

### 3.2. Data Sampling and Collection

Random sampling and snowball sampling techniques were used to collect the data. It was impossible for us to go to Wuhan due to quarantine during the outbreak. We were quarantined by ourselves. To avoid this hindrance on data collection, we decided to collect data online. We sent our questionnaire to the people of Wuhan and Anhui provinces. Data was collected during the COVID-19 quarantine period, which has been in place for almost one month and continued during the research. Most of the participants were Chinese; however, we also included foreigners living in Wuhan and Anhui because of their presence. However, due to various countries’ evacuation policies, we decided to exclude the foreigners’ data. Due to the mixed participants of our study, we used two versions of the questionnaire to overcome any language barrier. We used an English version for data collection from foreigners and a Chinese version for the domestic population in Wuhan and Anhui.

To increase interest and willingness of participants we sent them HONGBAO (lucky money) to the group owners of Wechat through Wechat (only owners, not the participants). We collected data from 704 people (still ongoing, the corresponding author is willing to help any researchers with future research). In total, the participants included 315 men and rest were women (we aimed to obtain participants equally from both sexes). Due to online foreigners’ evacuation during data collection, we excluded 21 records during analysis and used 683 valid samples for analysis. After the evacuation policy implementation, we decided to stop the collection from foreigners. The demographic characteristics of our research data are provided in Table 1. Moreover, the itemized sources of constructs used in the research are given in Table 2.

### 3.3. Statistical Analysis

**Figure 3:**
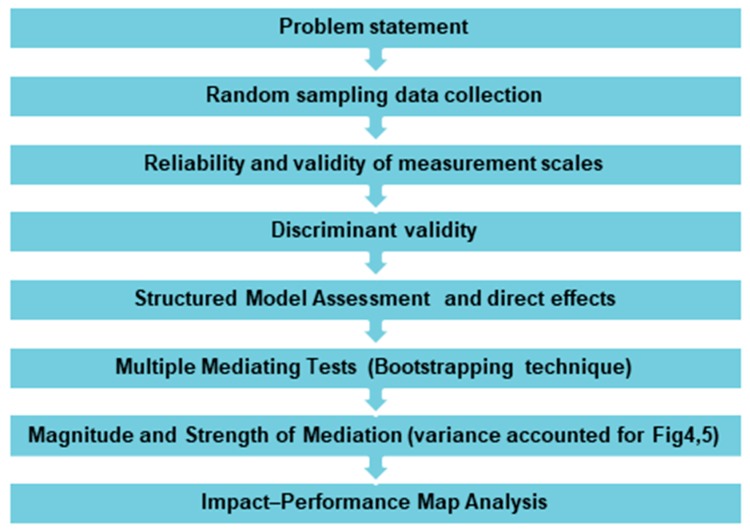
Steps in methodology.

The steps in our methodology are reported in Figure 3.

From 2013 onward, a noticeable increase in participation of partial least squares structural equation modeling has been reported [72]. If not familiar with the data type or if the data have a common factor or are composite-based, findings illustrate that use partial least squares (PLS) is the best choice for analysis [73]. Appraisal and review studies explained that in management research with multivariable analysis techniques, the application of partial least squares structural equation modeling (PLS-SEM) has been increased [74]. PLS-SEM is being increasingly used in investigative and theory-based research [75]. Use of PLS is increasing in different branches of management, especially research with one-variable-based techniques [76]. Past studies showed that PLS-SEM in different branches of management research have used multivariate analysis. As research in online user psychology is in its infancy and is not as developed as management research, the existing psychological studies about patients during pandemics during a sensitive time period (isolation)does not adequately explain the behavioral psychology of online users. As such, we applied PLS-SEM (smart PLS) in our research [74].

## 4. Results 

### 4.1. Measurement Model Assessment

To assess our measurement model, we verified the concurrent validity, discriminate validity, and composite reliability (CR) [77]. In addition to the square root values of the average variance extracted (AVE), we compared constructs to determine the discriminate validity [78]. Factor loading values should be greater than 0.70 [79]. For data validity and measurement, the value of the AVE should be greater than 0.5 [80], CR > 0.7 [78], and rho > 0.7 [81]. Table 3 provides the reliability and validity of our measurement scales and Table 4 provides the results of the Fornell–Larcker test used to check distinguished and divergent validity.

### 4.2. Structured Model Assessment

To create intervals of confidence and t-values, we used bootstrapping (4000 re samples) to check for imagined associations between the concerned constructs of the planned structured model. Streukens, S., et al. [82] stated that bootstrap replications can vary considerably from a minimum of 500 to a maximum of 5000.In other words, statistic inconsistency is checked using the inconsistency of data using bootstrapping, which is a nonparametric resampling method, instead of using parametric statements to check the accuracy of approximation [83]. Efron, B., et al. [84] proposed using more than 1000 bootstrap samples.

**Figure 4:**
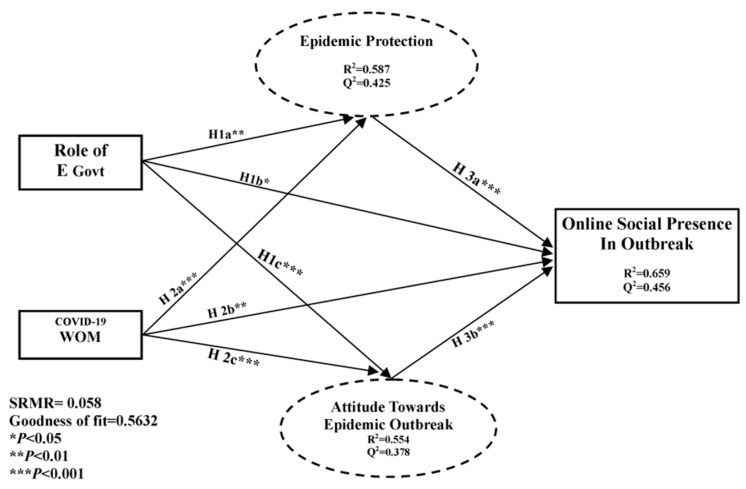
Structured model and direct effects.

The mediation effect is absent if the direct effect is not significant. Figure 1 illustrates the hypotheses testing of direct effects, which are also shown in Table 3. Table 3 also provides the fit statistics. Dependent variables indicate an important and positive precursor to their independent variables. Particularly, the role of E-Govt was a noteworthy predictor of epidemic protection. As can be seen from Figure 4 and Table 5, all of the hypotheses were supported [85] for the direct effect hypothesis at this step.

To check the importance of the structural path coefficients, we report the confidence interval [86]. These were supported because we did not add up zero values in any confidence interval [78]. At present, in standardized root mean square residual (SRMR) PLS path modeling, mostly model fit criteria are used. We checked the accuracy of the fit by using different tools like value of normed fit index (NFI), the non-normed fit index (NNFI), the comparative fit index (CFI), root mean square error of approximation (RMSEA), and SRMR. Values equal to or higher than 0.95 in NFI, NNFI, and CFI indicate the best fit. Sufficient adjustment was represented by RMSEA and SRMR with values less than 0.06 [87]. For comparatively good fit between the hypothesized model and observed data, a cut-off value near to 0.08 for SRMR and near 0.06 is best for RMSEA; Hu, L.T. et al. [88] stated that a zero value for SRMR indicates an ideal fit but if the value is smaller than 0.05,the fit is satisfactory fit [89]. We investigated the standards of the coefficient of determination (*R*^2^) to verify the predictive strength of our structure model. Collective consequences of exogenous contracts on endogenous constructs were indicated. The *R*^2^ of the endogenous latent variables is the vital decisive factor for this evaluation. This marker is used, from the perspective of a statistical model, to forecast future results or can be used to check the hypothesis on behalf of other related information. *R*^2^ also provides the results of the calculations and describes the practicality of the results [90]. Researchers can also use PLS procedure to check their model’s out-of-sample predictive power [91]. In-sample predictive power, we also refer to the *R*^2^ [92]. *R*^2^ varies from 0 to 1 and greater values show better descriptive power. Substantial, moderate, and weak descriptive powers are indicated by R^2^ values of 0.75, 0.50, and 0.25, respectively [93]. PLS-SEM is less dependent on the model fit concept compared to CB-SEM [79].

As recommended [74] RMSEA cut-off values equal to or less than 0.08 using modification in *R*^2^ report effect size (*f^2^*) indicated that the effect of our dependent variables on independent variables was very satisfactory.

### 4.3. Effect Size and Predictive Relevance

The effect of the exogenous latent construct on the endogenous latent construct having three possible answers, i.e., substantial, moderate, and weak, was found using theF^2^ effect size. The blindfold method was used to check the strength of the research model. Cohen’s *f*^2^ is an identical measure of effect size that also permits checking the local effect size, which is the effect of one variable compared with the multivariate regression model [94]. If the cross-validated redundancy (*Q*^2^) is higher than 0,then the model is related to predicting that factor [95]. We focused on in-sample prediction more, compared to out-sample prediction, prognostic significance Q^2^, and relative relevance Q^2^, which are alternatives for evaluating a model’s practical relevance, in addition to consulting *R*^2^ outcomes as a gauge of a model’s predictive capabilities [95]. *R*^2^, Q^2^, path coefficients, and the effect size (f^2^) are the decisive factors we use for evaluation. In addition to this evaluation, researchers are required to check the inner model for potential co linearity issues. If the constructs are interrelated, then results approximated by the inner model are considered biased [96]. A model’s predictive accuracy is decided by the *R*^2^. The *R*^2^ value also characterizes the combined consequence of exogenous variables on the endogenous variable(s). The effect ranges from 0 to 1. A value of 1 indicates complete predictive accuracy as can see in Table 6. 

Cohen’s f^2^ was calculated to check the effect size of each path model. When a construct was removed from the model, we calculated f^2^ while making no changes to *R*^2^. Researchers have to approximate two PLS path models for computing f^2^. The effect size of the omitted construct for a particular endogenous construct can be found by standard values: 0.02 for small, 0.15 for medium, and 0.35 for a large effect on the basis of the f^2^ value [96].

This discussion supports the use of our mediators and variables in the model. 

### 4.4. Multiple Mediating Effect Tests

In the PLS path model, mediator variables absorb the effect of an exogenous construct on an endogenous construct; this absorption of effect is known as meditation [96]. The mediation effect can be investigated using many tools, including PLS-SEM. Though researchers use an older method to determine the mediation effect in PLS-SEM, the procedure that identifies the effect of a precursor variable on the findings and results is judged by mediation; in other words, mediation considers transitional variables [97]. A mediating variable may have a transitional role in the association between dependent and independent variables, and engagement of this third variable is the important feature of the mediating effect [98]. Clarification and elaboration are the main effects of mediation [81]. As such, we included multi-mediation concepts in the results of social presence theory and for psychological aspects during outbreaks (see Table 7).

**Figure 5:**
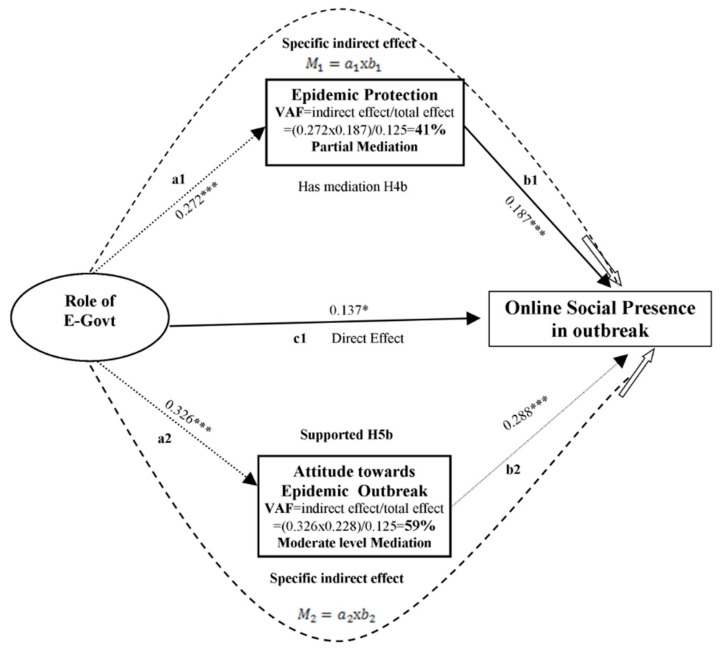
Magnitude of mediation of H4b and H5b.

**Figure 6:**
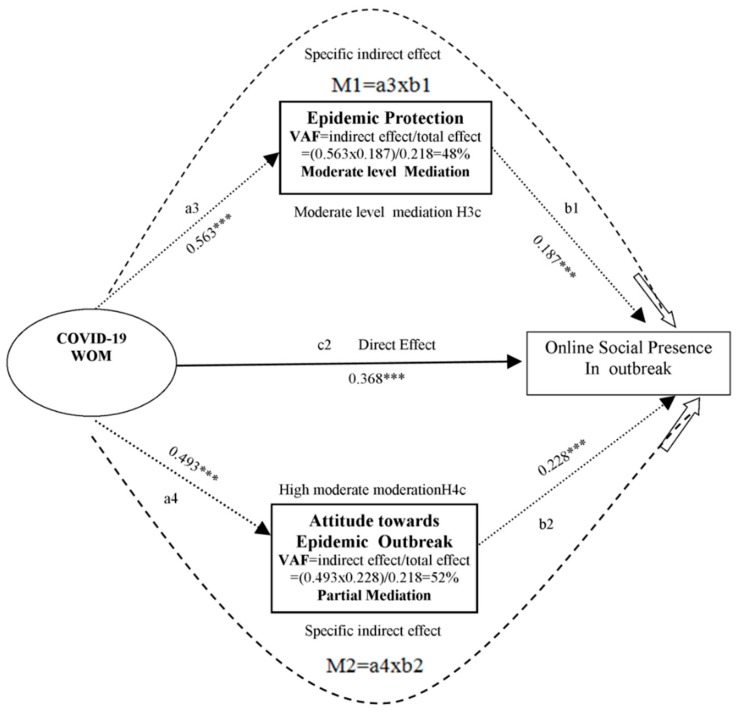
Magnitude of mediation of H3c and H4c

The results of the effects of exogenous variables (role of E-Govt and 2019-nCoV-WOM) on the endogenous variable (online social presence) with the mediating effect are provided in Table 7. Multiple mediation paths outcomes and potency (level) of mediation effects are demonstrated in Figure 5 and Figure 6. Precise indirect effects were investigated by bootstrapping techniques with the help of the bias correction technique. The two independent variables (role of E-Govt and2019-nCoV-WOM) affected online social presence; these effects support H1b, H2b, and H3c in that order, as mentioned above.

### 4.5. Magnitude and Strength of Mediation

To determine whether H4b, H5b, H3c, and H4c are supported, we used the proposals by Hair et al. [85]. To finalize conclusions about the mediation effect; we calculated the amount and magnitude of mediation. We incorporated the variance accounted for (VAF) method to calculate the strength of mediation (Figure 5 and Figure 6). If VAF is less than 0.2, there is no mediation; if VAF is less than or equal to 0.8, there is a partial mediation; if VAF is greater than 0.8, there is full mediation. The magnitude and strength of epidemic protection (H4b: a1b1) and attitude toward epidemic outbreak (H5b: a2b2) mediated the association between the role of E-Govt and online social presence (Figure 5). We found via comparison that epidemic protection has a partial mediation effect on the role of E-Govt and online social presence because the VAF value was more than 0.2, which indicated that there is a partial mediation effect. As such, H4b is supported. Attitude toward epidemic outbreaks mediated the association between 2019-nCoV-WOM and online social presence; the VAF value was greater than 0.2, which indicated the presence of mediation. Therefore, we hypothesized imaginary harmonizing partial mediation because the effects of 2019-nCoV-WOMwere considerable both directly and indirectly and their products were positive [99].

Hair et al. [85] Concluded that complementary and competitive mediation can be differentiated if direct and indirect effects are more prominent. The condition in which direct and indirect effects work in the same direction is called complementary mediation. This means the outcome of the direct and indirect effect is positive. The magnitude and strength of the mediation effect of epidemic protection (H3c: a3b1) and attitude toward epidemic outbreak (H4c: a4b2) mediating the association of 2019-nCoV-WOMand online social presence is shown in Figure 6. In comparison, the VAF value was higher than 0.2, which indicated the presence and effect of mediation. Due to the prominent direct and indirect effects of 2019-nCoV-WOM, the complementary partial mediation was also positive. The comparison showed that the association between 2019-nCoV-WOM and online social presence was mediated by attitude toward epidemic outbreak. The VAF value was higher than 0.2, which indicated partial mediation, also supporting the multi-mediation hypothesis [99].

### 4.6. Impact–Performance Map Analysis (IPMA)

IPMA, also called impact–performance map or priority map analysis, is a useful approach in PLS-SEM. IPMA adds facets and measurements that consider the scores of latent variables reporting the path coefficient [99].

**Figure 7:**
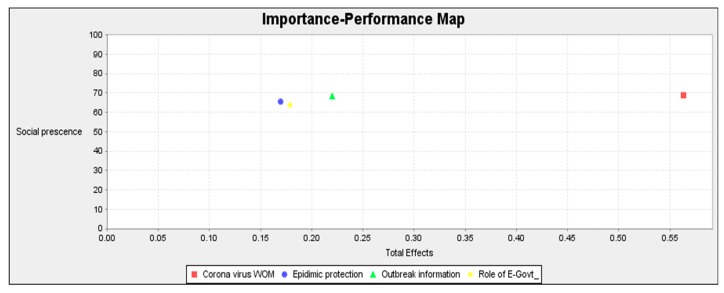
Impact performance map analysis (IPMA).

Approaches to determining the role of precursor constructs and their significance for management actions are offered by the PLS-SEM studies based on IPMA. IPMA compares the significance and recital (performance of the variables) [99]. Analysis dimensions are used to demonstrate the results of path coefficient approximation extended by IPMA in Figure 7. The advantage of IPMA is the validation of total effects and a representation of their significance in a construct with an average score that indicates their performance. Our main purpose with the construct was to find the most significant component in the construct.

Online ratings are associated with higher enjoyment than negative reviews [100]. To the best of our knowledge, the broader tasks and household behaviors of the community and family members in social media and role of E-Govt have been relatively under-examined.

## 5. Discussion

We used an online questionnaire and proposed a unique conceptual model and multi-mediation model to achieve the objectives of our study. We constructed eight hypotheses for the direct effects (H1a, H1b,H1c,H2a,H2b,H2c,H3a, and H3b) and four hypothesis (H4b,H5b,H3c, and H4c) for the mediation effect of our dual mediators, i.e., epidemic protection and attitude toward epidemic outbreak, with their indirect effect between the role of E-Govt and2019-nCoV-WOM on online social presence during outbreaks. Our results are supported by Mmijail et al. [101] who concluded that local government affects the attitude and decision-making process of people with their e-government platforms. The results of our study showed that role of E-Govt have a strong effect on the attitude of the public toward quarantine. Our study results also showed that public relationship directly influences positive WOM. Our study results are supported by Kim et al., who concluded that local government affects the social presence of community participants and the identified individuals’ attitudes and community [102]. Our study results revealed that attitude toward epidemic outbreak has a strong mediation effect between the role of E-Govt and online social presence during outbreaks, indicating that other governments and organizations can follow China’s safety model. The Chinese government allowed full opportunity to be online and for online users to promote hand washing and mask wearing during the COVID-19 outbreak.

As for as effect of 2019-nCoV-WOM and online social presence is concerned, our study findings are supported a the previous study [102] in which human voice and WOM were found to have a positive impact on social presence. Our findings showed that 2019-nCoV-WOM has a positive effect on online social presence.

## 6. Conclusions

Online social presence is increasing worldwide. Social media has become increasingly important, especially for COVID-19 information. In this study, we determined the impact of the role of e-government and COVID-19 word of mouth on online social presence. We estimated the mediation impact of epidemic protection and attitude toward epidemic outbreaks on online social presence. The key results showed that the role of e-government and COVID-19 word of mouth positively impacted online social presence. Similarly, epidemic protection and attitude toward epidemic outbreak showed positive mediation impact on online social presence. From estimated results, we outline some implications and policy suggestions:

During quarantine, people have more free time to participate in social media, which increases their desire to be present online. For themselves and society, they want to participate in disease protection and to provide suggestions to perform positively during the difficult time caused by COVID-19 quarantine. People can obtain basic information and protection measures from e-government and obtain specifics about the issue. 

For practical implementations during epidemic outbreaks, the results suggest that the health authorities and government should pay more attention to managing the attitude toward outbreaks and its relationship with the role of e-government. People’s perceptions about the government will help build their willingness toward long-term pandemic control.

## 7. Research Limitations and Future Directions

Some limitations were unavoidable in our study. We used two sampling techniques: random sampling and snowball sampling; future research can be improved using different kind of sampling and data collection techniques. The second major limitation was the use of single type of role of E-Govt. The reason behind this limitation was the ongoing quarantine, so it was impossible to compare the relationship of 2019-nCoV-WOM in online social presence with offline discussion because personal meetings, face-to-face contact, physical interviews, etc. were prohibited. However, future research can be improved using different types of research variables, using web scraping and web mining of top trends complete protection analyses. However, our findings can be implemented to improve online social presence and increase emotive protection during epidemic quarantine periods. Fourth, the data were collected online. Therefore, we were unable to gauge the respondents’ responses during data collection, although people were very motivated to share their answers to the questionnaire because of the involvement of the role of the government. More adequate research can be conducted by expanding the study area, e.g., people from other countries. Our study was limited to two provinces because as of6 March, 2020, these two provinces were still under quarantine. The study could also be further improved by focusing on recovered patients and comparing different countries affected by COVID-19 using the proposed research model. Further research on special issues is highly encouraged in other countries that have different isolation facilities, e.g., free internet, quiz competitions for children, etc., on the basis of the theoretical background, web scraping, and trends.

## Figures and Tables

**Table 1:** Demographics of study participants.

Classification		Frequency (*n*)	Percentage
Sex	Male Female	315 368	46.12% 53.87%
Marital status	Married Unmarried Divorced	309 356 18	45.24% 52.12% 2.63%
Age	Under 18 18–30 31–40 41–50 Above 50	27 259 225 143 29	3.95% 37.92% 32.94% 20.93% 4.24%

**Table 2:** Scale development.

Construct	Items and Sources
Role of E-Govt	Efforts of E-Govt, trust in E-Govt, support of E-Govt [26]
2019-nCoV-WOM	Information,countries’ status, 2019-nCoV- plan [68]
Epidemic protection	Hand wash, mask, motivation to protect [69,70]
Attitude toward epidemic outbreak	Willingness to quarantine, health psychology, doctors’ advice [71]
Online Social presence in outbreak	More present in quarantine, present for social support, present to discuss COVID-19

**Table 3:** Reliability and validity of measurement scales.

Construct	Item	Outer Loading	Mean	SD	α	CR	AVE
Role of E-Govt	E-Govt 1	0.921	5.045	1.376	0.8	0.883	0.717
	E-Govt 2	0.935	5.104	1.155			
	E-Govt 3	0.932	5.125	1.144			
2019-nCoV-WOM	CONV-1	0.749	5.557	0.853	0.921	0.95	0.864
	CONV-2	0.895	5.509	0.994			
	CONV-3	0.89	5.402	0.947			
Epidemic protection	E-P 1	0.901	5.255	1.026	0.847	0.908	0.767
	E-P 2	0.913	5.321	1.077			
	E-P 3	0.809	5.227	0.807			
Attitude toward epidemic outbreak	ATOB 1	0.844	5.427	0.924	0.806	0.886	0.721
	ATOB 2	0.846	5.364	0.856			
	ATOB 3	0.857	5.469	1.058			
Online social presence in Outbreak	S-P 1	0.877	5.254	0.902	0.817	0.891	0.732
	S-P2	0.855	5.38	0.98			
	S-P 3	0.835	5.305	0.818			

Note: E-Govt, role of E-government; CONV, COVID-19 word of mouth; E-P, Epidemic protection; S-P, online social presence during outbreak.SD, α, CR, and AVE are standard deviation, Cronbach’s α, composite reliability, and average variance extracted, respectively.

**Table 4:** Discriminant validity.

	ATOB	2019-nCoV-WOM	E-Govt	S-p	E-P
ATOB	**0.849**				
2019-nCoV-WOM	0.701	**0.847**			
E-Govt	0.640	0.637	**0.93**		
S-P	0.710	0.753	0.636	**0.856**	
E-P	0.727	0.737	0.631	0.711	**0.876**

Note: Values in bold indicate square root of average variance extracted (AVE), which must be higher than the values in the column to confirm validity.

**Table 5:** Structured model.

	Relationship	Direct Effect	t-Value	Decision	F^2^
H1a	E-Govt→ E-P	0.272	8.075	Supported	0.107
H1b	E-Govt→ S-p	0.137	3.317	Supported	0.028
H1c	E-Govt→ ATOB	0.326	8.68	Supported	0.142
H2a	conv19-WOM→E-P	0.563	16.353	Supported	0.456
H2b	conv19-WOM→S-P	0.368	8.34	Supported	0.149
H2c	conv19-WOM→ATOB	0.493	12.674	Supported	0.323
H3a	E-P→S-P	0.187	4.359	Supported	0.037
H3b	ATOB→S-P	0.228	5.835	Supported	0.059

**Table 6:** Effect size and predictive relevance.

Endogenous Variables	Q^2^	*R* ^2^	Exogenous Variables	Effect Size f^2^
E-P	0.425	0.587	E-Govt 2019-nCoV-WOM	0.107 0.456
ATOB	0.378	0.554	E-Govt 2019-nCoV-WOM	0.142 0.323
S-P	0.456	0.659	E-Govt 2019-nCoV-WOM E-P ATOB	0.028 0.149 0.037 0.059

Note: E-P, S-P, and ATOB are dependent variables; E-Govt and 2019-nCoV-WOM are independent variables.

**Table 7:** Mediation analysis.

	Mediation Path	Specific Indirect Effect	T-value	*p*-value	Total Effect
**H** **4** **b**	**E-Govt**→ **E-P**→**S-P**	0.051	3.951	0.000	0.125*** (7.320)
**H** **5b**	**E-Govt**→**ATOB**→**S-p**	0.074	5.189	0.000	
**H** **3c**	**2019-nCoV-WOM**→**E-P**→**S-P**	0.106	4.093	0.000	0.218*** (7.224)
**H4c**	**2019-nCoV-WOM**→**ATOB**→**S-P**	0.113	5.262	0.000	

Note: E-P and ATOB are mediating variables; E-Govt and 2019-nCoV-WOM are independent variables. S-P is dependent variables for this table. *** indicates strong mediation.
